# New and Summary Mode of Creating Lawyers and Doctors

**Published:** 1847-05

**Authors:** 


*New and Summary mode of creating Lawyers and Doctors.*

Under this caption the Editor of the St. Louis Med. and Surg. Journal, published the following act recently passed by the Missouri Legislature, and comments thereon.—Ed. Buff. Jour.

Section 7. Every person, or co-partnership of persons, in this State, who shall follow the practice of the law for a livelihood, in whole or in part, IS HEREBY DECLARED TO BE A LAWYER ; and every person, or co-partnership of persons, in this State, who shall follow the practice of medicine for a livelihood, in whole or in part, is hereby declared to BE A PHYSICIAN.

Our law schools and medical colleges may now close their doors forever, and our young men may no longer trouble themselves with the dry and musty details of the law, or with the tedious and uninteresting process of attending medical lectures. For, who will be hereafter stupid enough to go through a laborious course of study, for the purpose of obtaining a diploma, when, by the solemn act of the Legislature of a sovereign State, he is declared to be a lawyer or doctor, solely by hanging out his shingle ! ! Verily we live in a remarkable age, and in a wonderful country ! Hitherto sages and philosophers have been unable to discover a royal road to learning, or the act of effecting by the simple act of volition, that, which under ordinary circumstances, it would require a life time to accomplish ; but what the hoary headed sires of antiquity could not do, has been made plain and easy by the wiseacres of the Missouri

Legislature. We reccollect in our school-boy days, to have read of a certain Roman general who gained infamous notority by attempting to confer the honors of knighthood on his horse, but it never entered into our poor philosophy to dream that it was reserved for modern "*progressive democracy*," to confer the honors of literary institutions, indiscriminately on all, without reference to moral or intellectual qualification. O, tempora! O, mores!!!

